# Citizen science reveals landscape-scale exposures to multiazole-resistant *Aspergillus fumigatus* bioaerosols

**DOI:** 10.1126/sciadv.adh8839

**Published:** 2023-07-21

**Authors:** Jennifer M. G. Shelton, Johanna Rhodes, Christopher B. Uzzell, Samuel Hemmings, Amelie P. Brackin, Thomas R. Sewell, Asmaa Alghamdi, Paul S. Dyer, Mark Fraser, Andrew M. Borman, Elizabeth M. Johnson, Frédéric B. Piel, Andrew C. Singer, Matthew C. Fisher

**Affiliations:** ^1^ MRC Centre for Global Infectious Disease Analysis, Department of Infectious Disease Epidemiology, Imperial College London, London, UK.; ^2^ UK Centre for Ecology & Hydrology, Wallingford, Oxfordshire, UK.; ^3^ Department of Medical Microbiology, Radboud University Medical Center, Nijmegen, Netherlands.; ^4^ School of Life Sciences, University of Nottingham, Nottingham, UK.; ^5^ Faculty of Science, Department of Biology, Al-Baha University, Al-Baha, Saudi Arabia.; ^6^ UK National Mycology Reference Laboratory, National Infections Service, Public Health England, Science Quarter, Southmead Hospital, Bristol, UK.; ^7^ MRC Centre for Medical Mycology, University of Exeter, Exeter, UK.; ^8^ NIHR HPRU in Environmental Exposures and Health, Department of Epidemiology and Biostatistics, Imperial College London, London, UK.

## Abstract

Using a citizen science approach, we identify a country-wide exposure to aerosolized spores of a human fungal pathogen, *Aspergillus fumigatus*, that has acquired resistance to the agricultural fungicide tebuconazole and first-line azole clinical antifungal drugs. Genomic analysis shows no distinction between resistant genotypes found in the environment and in patients, indicating that at least 40% of azole-resistant *A. fumigatus* infections are acquired from environmental exposures. Hotspots and coldspots of aerosolized azole-resistant spores were not stable between seasonal sampling periods. This suggests a high degree of atmospheric mixing resulting in an estimated per capita cumulative annual exposure of 21 days (±2.6). Because of the ubiquity of this measured exposure, it is imperative that we determine sources of azole-resistant *A. fumigatus* to reduce treatment failure in patients with aspergillosis.

## Teaser

Citizen science shows widespread exposure to aerosolized fungal spores that have evolved resistance to clinical drugs.

## INTRODUCTION

The cosmopolitan mold *Aspergillus fumigatus* is a key saprotrophic fungus in the breakdown of a range of plant and soil organic matter (*1*). However, it is also an opportunistic pathogen capable of causing a spectrum of chronic and acute life-threatening diseases in humans. The widespread occurrence of resistance to first-line clinical azole drugs in environmental isolates of the mold, alongside molecular epidemiology linking azole-resistant *A. fumigatus* (AR*Af*) sourced from patients and the environment, argues that a substantial burden of treatment failure is due to the evolution of resistance in the fungus resulting from environmental exposure to azole-based fungicides (*2*). The number of patients in the United Kingdom (UK) presenting with infections that are resistant to one or more of the clinical azoles is increasing in diverse patient groups (*3*, *4*), matching other worldwide reports (*5*, *6*). The substantially elevated case fatality rates where invasive aspergillosis is caused by AR*Af* (*7*, *8*) further highlight the importance and breadth of this emerging problem.

The widespread use of broad-spectrum agricultural fungicides, founded on the same demethylase inhibitor (DMI) chemistry as the clinical azoles, has long been argued to drive the evolution of environmental resistance (*9*). This hypothesis has found broad support from surveillance demonstrating environmental hotspots of *A. fumigatus* growth alongside high frequencies of resistance where this saprotrophic mold has the potential to grow in the presence of agricultural DMIs (*10*). Yet, despite its increasingly wide detection in the environment worldwide (*10*, *11*), little is known about the extent to which humans are exposed to AR*Af*. The mold is adapted to airborne dispersal and most humans inhale large numbers of viable spores every day (*1*, *12*). Because of the potential clinical consequences of their inhalation, occupational exposures to these spores are legislated for in countries such as the UK, especially in green-waste recycling and composting processes that have the potential to generate high levels of inocula (*13*). However, occupational monitoring does not include assessing exposure to AR*Af.* Surveillance has shown hotspots of environmental resistance in both homes (*11*) and industrial compost (*14*), urban environments (*15*), greenhouses (*16*), and horticultural products (*17*). Nonetheless, there is little insight into population-wide exposures of at-risk individuals to aerosolized AR*Af* occurring beyond these heterogeneous environmental foci.

Given the dynamic nature of the atmosphere and the potential for a season to affect the biology of *A. fumigatus* spore production in many countries, meaningful assessment of human exposure to AR*Af* needs to be undertaken at a population level in a cost-effective manner that spans yearly seasonal variation. To meet this need, we recruited and led a citizen science project (*18*) to simultaneously sample and enumerate live airborne spores of *A. fumigatus* across multiple time points throughout the year and at a scale that reflected the exposure of the UK’s population. Cultured isolates of the mold were then tested for susceptibility to a panel of agricultural and medical triazole drugs to determine their resistance phenotype. Subsequently, genome sequencing and phylogenomic analysis were used to determine relationships between these, and clinical and environmental triazole-resistant *A. fumigatus* isolates collected from the UK between 2005 and 2017 (*2*).

## RESULTS AND DISCUSSION

**Fig. 1.**
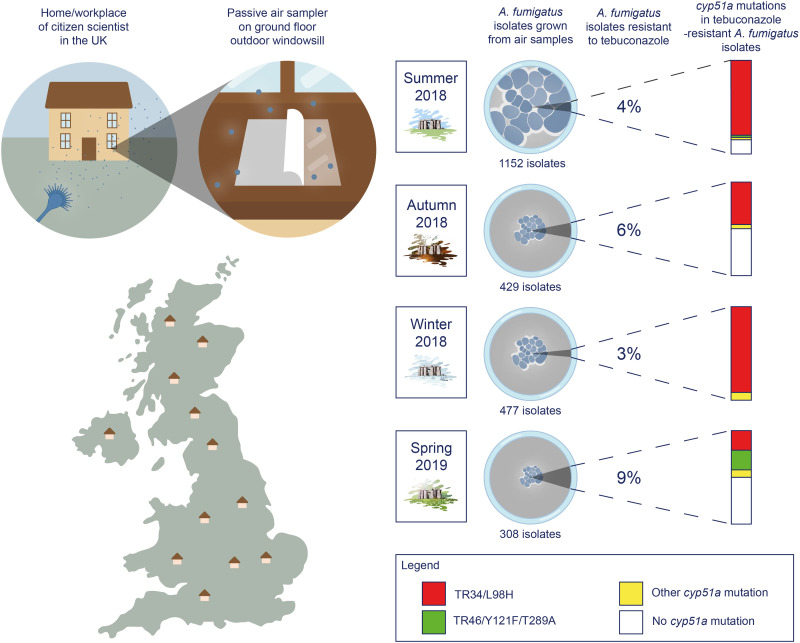
Infographic detailing the collection of air samples from outdoor ground floor windowsills of homes and workplaces across the UK on four solstice/equinox dates between summer 2018 and spring 2019. *A. fumigatus* isolates were cultured from air samples and tested for susceptibility to the agricultural fungicide tebuconazole, and tebuconazole-resistant isolates had their *cyp51A* promoter and coding regions sequenced to determine alleles responsible for azole resistance.

**Fig. 2.**
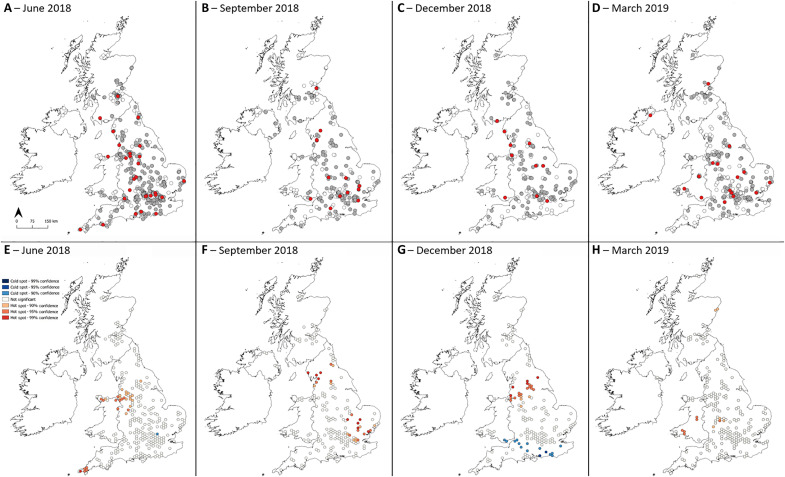
Maps and clusters showing locations across the UK where air samples were collected during four sampling rounds. Samples were collected on (**A**) 21 June equinox 2018, (**B**) 24 September solstice 2018, (**C**) 21 December equinox 2018, and (**D**) 20 March solstice 2019. White dots indicate samples that did not grow *A. fumigatus*, gray dots indicate samples that grew tebuconazole-susceptible *A. fumigatus*, and red dots indicate samples that grew tebuconazole-resistant *A. fumigatus*. Hotspots of azole-resistant *A. fumigatus* with 90, 95, and 99% confidence according to Getis-Ord Gi* cluster detection analysis for sampling rounds on (**E**) 21 June 2018, (**F**) 24 September 2018, (**G**) 21 December 2018, and (**H**) 20 March 2019.

Citizen scientists used simple passive air samplers to collect airborne spores of *A. fumigatus* synchronously across a 6- to 10-hour time period on the days matching the northern hemisphere seasonal equinoxes and solstices between 2018 and 2019 (Fig. 1). This activity resulted in a total of 1894 air samples being collected that, while being clustered owing to greater sample collection in areas of high population density, achieved a near UK-wide distribution at each time point (Fig. 2, A to D). These air samples were used to inoculate growth media, and then incubated at 43°C, a temperature that is highly selective for *A. fumigatus*. Of the screened air samples, 919 (49%) yielded a combined 2366 *Aspergillus* colonies. Of these colonies, secondary screening on media containing 6 mg/liters of the commonly used agricultural fungicide tebuconazole (TEB) identified 111 TEB-resistant isolates, comprising 4.7% of the total isolates recovered across all seasons (Table 1). This cumulative frequency is similar to that (~4%) measured at the Rothamsted Research station in 2016 suggesting an incidence that is relatively stable across recent years (*19*). Of the TEB-resistant isolates, 12 failed to sequence using the *cyp51A* promoter and coding region primers and were re-identified by matrix-assisted laser desorption/ionization–time-of-flight (MALDI-TOF) mass spectrometry (MS) as the related species *Aspergillus lentulus* (*n =* 10) and *Aspergillus nidulans* (*n =* 2). For the 99 TEB-resistant isolates confirmed to be *A. fumigatus*, clinical breakpoints then showed that 85 (86%) were resistant to itraconazole (ITZ), the first-line clinical drug for chronic infections, 63 (64%) were resistant to voriconazole (VCZ), the first-line agent for invasive infections, 18 (18%) were resistant to posaconazole (PCZ), and 82 (83%) were resistant to isavuconazole (ISZ) (Table 2 and table S1). Thus, there was clear cross-resistance between the agrochemical TEB and the medical azole antifungals, as reported elsewhere (*10*). Notably, 50 (51%) of AR*Af* were resistant to three medical azoles and 14 (14%) were resistant to all tested medical azoles (Table 2 and table S1), identifying a UK-wide aerosolized exposure to drug-resistant variants of this pathogen.

**Table 1.** The number of air samples collected, the number of air samples that grew *A. fumigatus*, and the number of samples that grew azole-resistant *A. fumigatus* (AR*Af*) across the four air sampling rounds.

Sampling round	Number of air samples collected	Number of air samples that grew *A. fumigatus* (% of samples)	Number of *A. fumigatus* isolates grown from air samples	Average number of *A. fumigatus* isolates grown per air sample	Number of air samples that grew AR*Af* (% of samples)	Number of AR*Af* isolates (% of *A. fumigatus*)
Summer	712	408 (57)	1152	2.8	30 (4)	42 (4)
Autumn	398	190 (48)	429	2.3	14 (4)	26 (6)
Winter	320	152 (48)	477	3.1	12 (4)	15 (3)
Spring	464	169 (36)	308	1.8	17 (4)	28 (9)
**Total:**	**1894**	**919 (49)**	**2366**	**2.6**	**73 (4)**	**111 (5)**

**Table 2.** Breakdown of 99 tebuconazole-resistant *A. fumigatus* according to cross-resistance to four medical azoles: ITZ, VCZ, PCZ, and ISZ. Dots indicate resistant minimum inhibitory concentrations according to clinical breakpoints, which were >1 mg/liter for ITZ, VCZ, and ISZ and >0.25 mg/liter for PCZ.

Sampling round	ITZ	VCZ	PCZ	ISZ	Number of AR*Af* isolates
Summer	∙	∙	∙	∙	10
	∙	∙		∙	23
	∙		∙	∙	3
					3
Autumn	∙	∙	∙	∙	3
	∙	∙		∙	13
	∙		∙	∙	1
	∙			∙	4
					3
Winter	∙	∙	∙	∙	1
	∙	∙		∙	2
	∙			∙	7
					1
Spring	∙	∙		∙	8
	∙			∙	6
		∙		∙	1
					9


To examine the genetic basis of the TEB-resistant phenotype, we genotyped the canonical locus that confers azole-resistance, the sterol-demethylase gene *cyp51A* (*20*, *21*). Environmental azole resistance in *A. fumigatus* is most commonly due to within-gene point mutations in *cyp51A* that are twinned with expression–up-regulating tandem repeats (TRs) in the promoter region (*22*). Genotyping confirmed that the most common aerosolized resistance-associated polymorphisms were TR_34_/L98H (59%) and TR_46_/Y121F/T289A (6%). We further found that 30% of TEB-resistant isolates did not contain any polymorphisms in the *cyp51A* promoter or coding regions suggesting that the existence of alternative resistance mechanisms as has previously been noted (table S1) (*23*). This is of concern in the clinical setting as only the two former mutations are picked up by commercially available polymerase chain reaction (PCR) diagnostic methods, although previously unidentified mutations leading to resistance will be identified by phenotypic testing of minimum inhibitory concentration (MIC) providing that there is an isolate available to test.

**Fig. 3.**
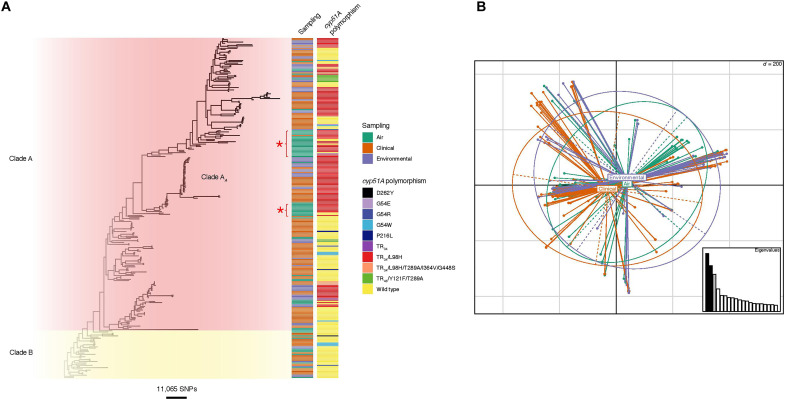
Phylogenetic and population genetic analyses indicating isolates sampled from air are not genetically distinct from the wider *A. fumigatus* population. (**A**) Unrooted maximum likelihood phylogenetic tree [constructed in RAxML using genome-wide single-nucleotide polymorphisms (SNPs)] showing the sampling type and *cyp51A* polymorphisms. Isolates sampled from air are found throughout the phylogeny, with two newly detected clusters of aerosolized isolates indicated by red stars. (**B**) Principal components analysis indicates genetic identity of isolates, with air sampled isolates broadly deriving from the same wider *A. fumigatus* population.

Subsequently, we determined the extent to which aerosolized AR*Af* matched those previously recovered and sequenced from UK terrestrial environments and patient cohorts by sequencing the genomes of 62 *A. fumigatus* isolates that were randomly selected to represent the phenotypes that were recovered from the summer 2018 air sampling round (21 wild-type; 41 TEB-resistant). These data were then combined with those resulting from prior UK-wide genomic surveillance (*2*) in phylogenetic analysis. The resulting tree showed that the aerosolized isolates were broadly distributed throughout the UK phylogeny (Fig. 3A) and were drawn from both previously described clades “A” [which contains the majority of AR*Af* (*2*)] and “B” (which is mainly sensitive to azole fungicides). Principal components analysis (PCA) (Fig. 3B) corroborated our conclusion that there was no clear differentiation between the aerosolized isolates collected in the present study as compared to those previously obtained from clinical and terrestrial sources, and nucleotide diversity (π) tests showed that the observed genetic diversity separating these groups was not significantly different (one-tailed *t* test *P* < 0.12591). We did, however, note two clusters of aerosolized isolates that were largely unrepresented in our previous surveillance, suggesting a temporally dynamic aspect to the UK population genetic structure of this fungus (Fig. 3A).

When mapped against the *A. fumigatus* reference genome *Af*293, each pair of isolates across the combined dataset were, on average, separated by 24,000 single-nucleotide polymorphisms (SNPs), a figure that was marginally lower (23,250 SNPs) when considering only aerosolized isolates. Notably, one genotype of an aerially sourced AR*Af* isolates from this study grouped within a previously identified UK-wide clonal subclade of genotypes, clade A*_A_*. Isolates in this clade all bear the hallmark TR_34_/L98H resistance allele and are widely found in the environment and in patients with aspergillosis. From these genomic data, we concluded that the genotypes of AR*Af* recovered from the UK aerobiome are largely (but not exclusively) representative of those azole-resistant genotypes recovered from patients. Moreover, these data indicate that ~40% (58 of 150 sequenced genomes) of AR*Af* infections are acquired from environmental exposures owing to the clinically derived isolates bearing the hallmark alleles that characterize environmental AR*Af*.

We next sought to explain the spatial occurrence of *A. fumigatus* and AR*Af* using logistic regressions to determine which cardinal environmental variables [season, maximum daily temperature, land cover classification, and proximity to the nearest industrial composting facility with open windrow or outdoor activity (OW/OA)] affected their likelihood. Individually, each variable was found to have a significant effect on whether a sample grew *A. fumigatus* (season χ^2^ = 50.3, df = 3, *P* < 0.01; maximum daily temperature χ^2^ = 29.3, df = 1, *P* < 0.01; land cover classification χ^2^ = 17.4, df = 7, *P* = 0.01; proximity to the nearest OW/OA composting facility χ^2^ = 15.1, df = 1, *P* < 0.01). The model with the lowest Akaike information criterion (AIC) included season, maximum daily temperature, and proximity to the nearest OW/OA composting facility, in which season and proximity to the composting facility had a significant effect on mold growth (Table 3). None of the environmental variables had a significant effect on whether the sample grew AR*Af*.

**Table 3.** Odds ratios, confidence intervals, and *P* values calculated for independent variables included in a logistic regression model using air samples collected in the United States (*n* = 1894) to explain whether a sample grew *A. fumigatus*. Significant results (*P* ≤ 0.05) are highlighted in bold.

Independent variable	Odds ratio (95% CI)	Pr(>|z|)
Sampling round		
Summer (baseline)		
Autumn	**0.75 (0.57–0.99)**	**0.05**
Winter	0.94 (0.57–1.57)	0.82
Spring	**0.53 (0.37–0.75)**	**<0.01**
Maximum daily temperature at sampling location on sampling date	1.04 (0.99–1.09)	0.13
Proximity of sampling location to the nearest OW/OA composting facility	**0.99 (0.99–0.99)**	**<0.01**

In the negative binomial regression determining which environmental variables affected the number of *A. fumigatus* colonies grown, sampling round (χ^2^ = 35.9, df = 3, *P* < 0.01) and proximity of sampling location to the nearest OW/OA composting facility (χ^2^ = 10.2, df = 1, *P* < 0.01) showed significant associations. Samples collected in autumn and spring grew significantly reduced numbers of *A. fumigatus* colonies compared to samples collected in summer (both *P* < 0.01) and increasing the distance of sampling location from the nearest OW/OA composting facility was significantly associated with a reduction in the number of *A. fumigatus* colonies grown from samples (*P* < 0.01). None of the environmental variables had a significant effect on number of AR*Af* colonies grown, although it is worth noting that a greater number of AR*Af*-positive samples may be required to identify such associations than were obtained in this study.

Average nearest neighbor (ANN) tests found sampling locations in each sampling round to be clustered (Table 4) and Getis-Ord Gi* spatial clustering analysis detected hotspots (high prevalence) and coldspots (low prevalence) of airborne AR*Af* in each sampling round (Fig. 2, E to H). The detected clusters were not geographically stable between sampling rounds.

**Table 4.** Results for average nearest neighbor tests run for each sampling round separately and all sampling rounds together.

	Sampling round				
	Summer	Autumn	Winter	Spring	All
Number of samples	712	398	320	463	1893
Observed mean distance (m):	3,059	3,505	3,314	2,945	855
Expected mean distance (m):	10,544	14,103	15,728	13,075	6,467
Nearest neighbor ratio:	0.29	0.25	0.21	0.23	0.13
*z* Score:	−36.24	−28.68	−27.01	−31.89	−72.23
*P* value:	<0.00	<0.00	<0.00	<0.00	<0.00
Pattern	Clustered	Clustered	Clustered	Clustered	Clustered

In parallel to our aerosol sampling, a soil-sampling campaign was conducted by citizen scientists during the 2019 summer solstice (fig. S1), resulting in the recovery of a high burden of AR*Af* from garden soils totaling 736 TEB-resistant isolates (14% of total) from 246 locations (*11*). There were 46 participants from whom both soil and at least one air sample were collected (table S2). Of these, 23 (50%) grew AR*Af* colonies from either an air sample or soil sample, but only 3 (7%) grew AR*Af* colonies from both. Moreover, there were no locations from which AR*Af* was isolated from an air sample more than once. Together, these observations lead us to conclude that the locally dynamic nature of atmospheric flows means that our bioaerosol sampling strategy does not capture the presence of local AR*Af* soil hotspots. The corollary of this observation is that atmospheric mixing leads to the UK population being, on average, equally exposed to this bioaerosol. On the basis of our air sampling data and this assumption, we simulate that there are 63 (SD = 7.7) 8-hour time periods in the year where each individual across the UK is exposed to at least one viable AR*Af* spore, which equates to a cumulative exposure of 21 days (SD = 2.6 days) per year.

While our study only reports on aerosolized spores collected in the UK, this exposure is not restricted to the UK. Recruitment of citizen scientists attracted several participants from outside the UK (*24*) with air samples from Germany, France, and Netherlands growing viable AR*Af*. Fungal spores are readily dispersed across intercontinental scales (*25*), and our population genetic studies of *A. fumigatus* have shown that its worldwide distribution is unstructured, with no evidence for isolation-by-distance effects (*26*). Moreover, the usage of DMIs in agriculture is widely increasing where measured [see, e.g., (*27*)], and environmental azole-resistance has been detected in every country in which it was monitored (*28*) indicating that AR*Af* has achieved a global occurrence. Yet, to date, there has been no attempt to systematically measure population-wide exposures to AR*Af* more globally. This is changing, and in 2022 the pan-South American LatAsp (www.latasp.com) surveillance study commenced, mirroring the essential features of our citizen science campaign, with the aim of determining the continent-wide incidence of AR*Af*. Nonetheless, identifying the ecological hotspots that ultimately cause the miasma of azole-adapted mold that we document will require spatially downscaled approaches if suitable mitigation measures are to be developed.

## MATERIALS AND METHODS

### Culturing *Aspergillus fumigatus* from UK air samples

Air samples from which *A. fumigatus* isolates were cultured for this study were collected in four sampling rounds of a citizen science project that took place between June 2018 and March 2019 (*18*). The four sampling rounds took place on 21 June 2018 (summer solstice), 24 September 2018 (autumn equinox), 21 December 2018 (winter solstice), and 20 March 2019 (spring equinox). In total, 485 individuals collected 1894 air samples from England, Wales, Scotland, and Northern Ireland: 712 samples in summer, 398 samples in autumn, 320 samples in winter, and 464 samples in spring (Table 1).

Before the sampling date, citizen scientists posted two passive air samplers (measuring 6.8 cm by 8.0 cm) to collect *A. fumigatus* spores, which were MicroAmpTM clear adhesive films (Thermo Fisher Scientific, UK) cut in half. The sticky side of each sampler was exposed horizontally for 6 to 10 hours at approximately 1 m in height, on the sampling date, re-covered, and returned by post to the primary author. When an air sample was received, it was stored at room temperature until processing, which involved removing the cover and placing the sampler sticky-side down on a Sabouraud dextrose agar (SDA; Merck, Germany) plate containing penicillin (Merck, Germany) at 200 mg/liter and streptomycin (Merck, Germany) at 400 mg/liter. The plate was incubated at 43°C for 24 hours, the sampler was removed, and the plate was incubated for a further 24 hours at 43°C. *A. fumigatus* isolates were picked one at a time using a sterilized wooden toothpick into a tube containing mold preservation solution (MPS; 0.2% agar and 0.05% Tween 20 in dH_2_O) and stored at 4°C.

### Isolate screening for azole resistance

Isolates were screened for TEB resistance by pipetting 5 μl of MPS containing *A. fumigatus* spores onto an SDA plate containing TEB (6 mg/liter), and a subset of isolates (*n* = 250) were also tested using the Tebucheck protocol (*29*). The concentration of TEB (6 mg/liter) was chosen after testing the growth of 30 isolates with known *cyp51A* mutations on SDA supplemented with TEB (0, 4, 6, 8, and 16 mg/liter). Isolates able to grow at a TEB concentration of 6 mg/liter were tested for susceptibility to ITZ, VCZ, PCZ, and ISZ according to CLSI M38-A2, as described in Borman *et al*. (*30*). MICs were recorded as the lowest drug concentration at which no growth was observed, and MICs were considered resistant when they were >1 mg/liter for ITZ, VCZ, and ISZ and >0.25 mg/liter for PCZ which are the suggested clinical breakpoints.

### Identification of *A. fumigatus cyp51A* gene azole-resistance alleles

The promoter region of *cyp51A* was amplified using forward primer 5′-GGACTGGCTGATCAAACTATGC-3′ and reverse primer 5′-GTTCTGTTCGGTTCCAAAGCC-3′ and the following PCR conditions: 95°C for 5 min; 30 cycles of 98°C for 20 s, 65°C for 30 s, and 72°C for 30 s; 72°C for 5 min. The PCR reaction volume used was 50 μl: 10 μl of FIREPol DNA polymerase (Solis Biodyne, Estonia), 10 μl of forward primer (1.5 μM; Invitrogen, USA), 10 μl of reverse primer (1.5 μM; Invitrogen, USA), 18 μl of nuclease-free water (Merck, Germany), and 2 μl of DNA. Amplicons were visualized by gel electrophoresis and samples with visible bands were sent for sequencing using the forward primer. The coding region of *cyp51A* was amplified using forward primer 5′-ATGGTGCCGATGCTATGG-3′ and reverse primer 5′-CTGTCTCACTTGGATGTG-3′ and the following PCR conditions: 94°C for 2 min; 35 cycles of 94°C for 30 s, 60°C for 45 s, and 72°C for 45 s; 72°C for 5 min. The PCR reaction volume used was 50 μl: 0.2 μl of Q5 high-fidelity DNA polymerase (New England Biolabs, UK), 10 μl of Q5 reaction buffer (5X; New England Biolabs, UK), 0.5 μl of deoxynucleotide solution mix (40 μM; New England Biolabs, UK), 1 μl of forward primer (10 μM; Invitrogen, USA), 1 μl of reverse primer (10 μM; Invitrogen, USA), 35.3 μl of nuclease-free water (Merck, Germany), and 2 μl of DNA. Amplicons were visualized by gel electrophoresis and samples with visible bands were sent for sequencing in two segments using the primers 5′-CTGATTGATGATGTCAACGTA-3′ (*31*) and 5′-GATTCACCGAACTTTCAAGGCTCG-3′. Sequences were aligned using the Molecular Evolutionary Genetics Analysis (MEGA) software (Penn State University, USA) (*32*).

### Identification of isolates

Isolates that failed to sequence using the primers for the promoter and coding regions of *cyp51A* were identified using MALDI-TOF MS, as described by Fraser *et al*. (*33*).

### Whole-genome sequencing of *A. fumigatus* isolates

Genomic DNA (gDNA) was extracted from TEB-resistant isolates whose identity was confirmed as *A. fumigatus.* Isolates were revived from cryopreservation at −80°C by pipetting 20 μl into 25 cm^3^ NuncTM flasks containing SDA and incubating at 37°C for 48 hours. Spores were harvested by washing the surface of the SDA with 10 ml of phosphate-buffered saline plus 0.01% Tween 20, 1.8 ml of spore suspension was added to 2 ml FastPrep tubes (MP Biomedicals, USA), and tubes were centrifuged at 5000 rpm for 10 min. The supernatant was discarded and the pellet was resuspended in 300 μl of lysis solution and 1 μl of ribonuclease A from the MasterPureTM Complete DNA and RNA Purification Kit (Lucigen, USA). The kit protocol was followed, including an additional bead-beading step using a FastPrep-24TM instrument. Extracted gDNA was purified using a DNeasy Blood and Tissue Kit (Qiagen, Germany), and DNA concentration was measured using a Qubit fluorometer and a Qubit double-stranded DNA BR Assay kit (Thermo Fisher Scientific, UK). A NanoDropTM spectrophotometer (Thermo Fisher Scientific, UK) was used to assess DNA purity by checking that the ratio of absorbances at 260/230 nm and 260/280 nm was 1.8 to 2.0. Purified gDNAs were stored at −20°C before being sent to Earlham Institute (UK) where gDNA libraries were constructed, normalized, and indexed. Libraries were run on a NovaSeq 6000 SP v1.5 flow cell to generate 150–base pair paired-end reads. These data are deposited in the European Nucleotide Archive (ENA) under project accession number PRJEB51237.

All raw reads were quality-checked with FastQC v0.11.5 (Babraham Institute) and aligned to the reference genome Af293 (*34*) using BWA-MEM v0.7.8 (*35*) before conversion to sorted BAM format using SAMTools v1.3.1 (*36*). Variant calling was performed with GATK HaplotypeCaller v4.2.6.1 (*37*), excluding repetitive regions (identified by RepeatMasker v4.0.6), generating genomic variant call formats. Low-confidence variants were filtered providing that they met at least one of the parameters DP < 5, GQ < 50, MQ < 40, MQRankSum < −12.5, ReadPosRankSum < −8.0, SOR > 4.0. In addition, alternate variants must be present in at least 90% of the reads. SNPs were mapped to genes using vcf-annotator (Broad Institute).

Phylogenetic analysis was carried out on 62 TEB-resistant *A. fumigatus* isolates collected by air sampling in addition to 215 environmental and clinical *A. fumigatus* isolates with complete sampling history collected in the UK between 2005 and 2017 (*2*) (data available from ENA under project accession numbers PRJEB27135 and PRJEB8623). Whole-genome SNP data were converted to the presence/absence of an SNP with respect to the reference, and any SNPs identified as low confidence in the variant filtration step were assigned as missing. These data were converted to FASTA format. Maximum-likelihood phylogenies were constructed using rapid bootstrap analysis over 1000 replicates using the GTRCAT model of rate heterogeneity in RAxML v8.2.9 (*38*) to assess sequence similarity between isolates, and resulting phylogenies were visualized using ggtree v3.1.14 and Microreact (https://microreact.org/project/6NMrDobYGZnhmYnMSsHhC5-air) (fig. S2).

Genetic similarities were investigated using the hypothesis-free approaches PCA and discriminant analysis of principal components (*39*) using the R package adegenet v2.1.5 in R v4.1.0 (*40*). Nucleotide diversity tests were implemented in VCFtools v0.1.13 (*41*).

### Environmental variables thought to influence growth of *A. fumigatus*

Table 5 details the environmental variables that were ascertained for the sampling dates and locations based on the information provided by citizen scientists. The sampling round was used as a proxy for the season: 21 June 2018 (summer), 24 September 2018 (autumn), 21 December 2018 (winter), and 20 March 2019 (spring).

**Table 5.** Environmental variables obtained for air sampling locations and dates and the sources they were obtained from.

Environmental variables ascertained for sampling date and location	Source of information
Maximum daily temperature at sampling location on sampling date (°C)	Met Office HadUK-Grid dataset (*42*)
Land cover classification of sampling location (21 categories)	UKCEH Land Cover Map 2019 (*43*)
Distance of sampling location to nearest composter with OW/OA (m)	Composter locations obtained from Environment Agency, Scottish Environment Agency (SEPA) website (*44*), Natural Resources Wales website (*45*), and Northern Ireland Environment Agency website (*46*). Distances were calculated using package “geosphere” in R version 4.0.0.

### Generalized linear models

Generalized linear models (GLMs) run in R v4.0.0 were used to find associations between the environmental variables in Table 4 and (i) the likelihood of a sample growing *A. fumigatus* or AR*Af* and (ii) the number of *A. fumigatus* or AR*Af* colonies grown from a sample. Growth of *A. fumigatus* or AR*Af* from a sample was categorized as 0/1 and logistic regressions (“glm” function; family = “binomial”) were performed. Negative binomial regressions (NBRs) were run to find associations between environmental variables and numbers of *A. fumigatus* or AR*Af* colonies grown from a sample, as these data were overdispersed. The logistic regression models were run on 1894 samples, the *A. fumigatus* NBR was run on 919 samples that grew *A. fumigatus*, and the AR*Af* NBR was run on 73 samples that grew AR*Af*. A significant improvement on the null model, as determined by analysis of variance (ANOVA) using the chi-squared test, determined which environmental variables were included in the regression model. A reduced AIC score and a significant improvement on the null model were used to choose the regression model with the best fit. Results were considered significant when *P* ≤ 0.05.

### Spatial clustering analysis

ArcMap 10.7 was used to investigate the spatial distribution of all sampling locations and the samples that grew AR*Af*. Sample locations were georeferenced and projected using the British National Grid (EPSG:27700) coordinate reference system.

The ANN test was used to determine whether the sampling locations in each sampling round, and across all sampling rounds, were randomly distributed or spatially autocorrelated. This test measures the distance between each sampling location and the next closest sampling location and compares this to the distance that would be expected if the locations were randomly distributed throughout the study area (the UK). If the observed mean distance is less than the expected mean distance, then the spatial distribution of the observed data is considered spatially clustered. Alternatively, if the mean distance is greater than the expected distribution, then the features are considered dispersed. In both scenarios, ANN ratios were used to establish the type of spatial pattern (i.e., clustered, dispersed, or random), and associated *P* ≤ 0.05 was used to establish statistical significance.

A local indicator of spatial autocorrelation was used to detect and geographically identify hotspots of azole resistance in the UK. First, sampling locations were geospatially aggregated within a gridded hexagon structure (cell area, 115 km^2^). For each cell, the total number of collected samples and the total number of AR*Af* samples were extracted and used to calculate sample positivity (%). Cells with zero sample locations were removed before analysis. Then, a Getis Ord Gi* analysis was conducted to identify statistically significant hotspot cells based on sample positivity. This approach was used to generate local *z* scores for each cell, with statistically significant high and low values indicating hotspots and coldspots, respectively. For this analysis hotspot and coldspot cells were generated and grouped using three different significance values (90, 95, and 99%).

### Average exposure simulation analysis

For this analysis, we produced a matrix of 1,000,000 rows and 1,095 columns, where rows represented the number of individuals included in the simulation and columns represented the number of 8-hour time periods in 1 year (air samplers were exposed for an average of 8 hours on sampling days). Each column in the matrix was randomly assigned an AR*Af*-positive rate based on a truncated normal probability distribution—truncated to avoid negative values—with a mean of 5.78% and SD of 0.68%, to capture variability in AR*Af* prevalence between samples. The average of the column sums gave the number of AR*Af*-positive periods of 8 hours across the whole matrix. These AR*Af*-positive 8-hour periods were converted into total hours and then into days to give the average cumulative exposure of an individual in the UK to AR*Af* spores over the course of 1 year. This simulation assumed that any location could potentially be AR*Af*-positive at some point in time and did not account for spatiotemporal variability, which was detected in this study for *A. fumigatus* but not for AR*Af*.

## Notes

View/request a protocol for this paper from *Bio-protocol*.
