# Supplementary material for: Citizen science reveals landscape-scale exposures to multiazole-resistant Aspergillus fumigatus bioaerosols
Source: Sci Adv. 2023 Jul 21;9(29):eadh8839. doi: 10.1126/sciadv.adh8839 (PMC10361594; doi:10.1126/sciadv.adh8839)
Supplement: Supplementary file 1 — Figs. S1 and S2 Tables S1 and S2 [file sciadv.adh8839_sm.pdf]

Science Advances
AAAS

Supplementary Materials for

Citizen science reveals landscape-scale exposures to multiazole-resistant *Aspergillus fumigatus* bioaerosols

Jennifer M. G. Shelton *et al.*

Corresponding author: Jennifer M. G. Shelton, jenshe@ceh.ac.uk

Sci. Adv. **9**, eadh8839 (2023)
DOI: 10.1126/sciadv.adh8839

This PDF file includes:

Figs. S1 and S2
Tables S1 and S2

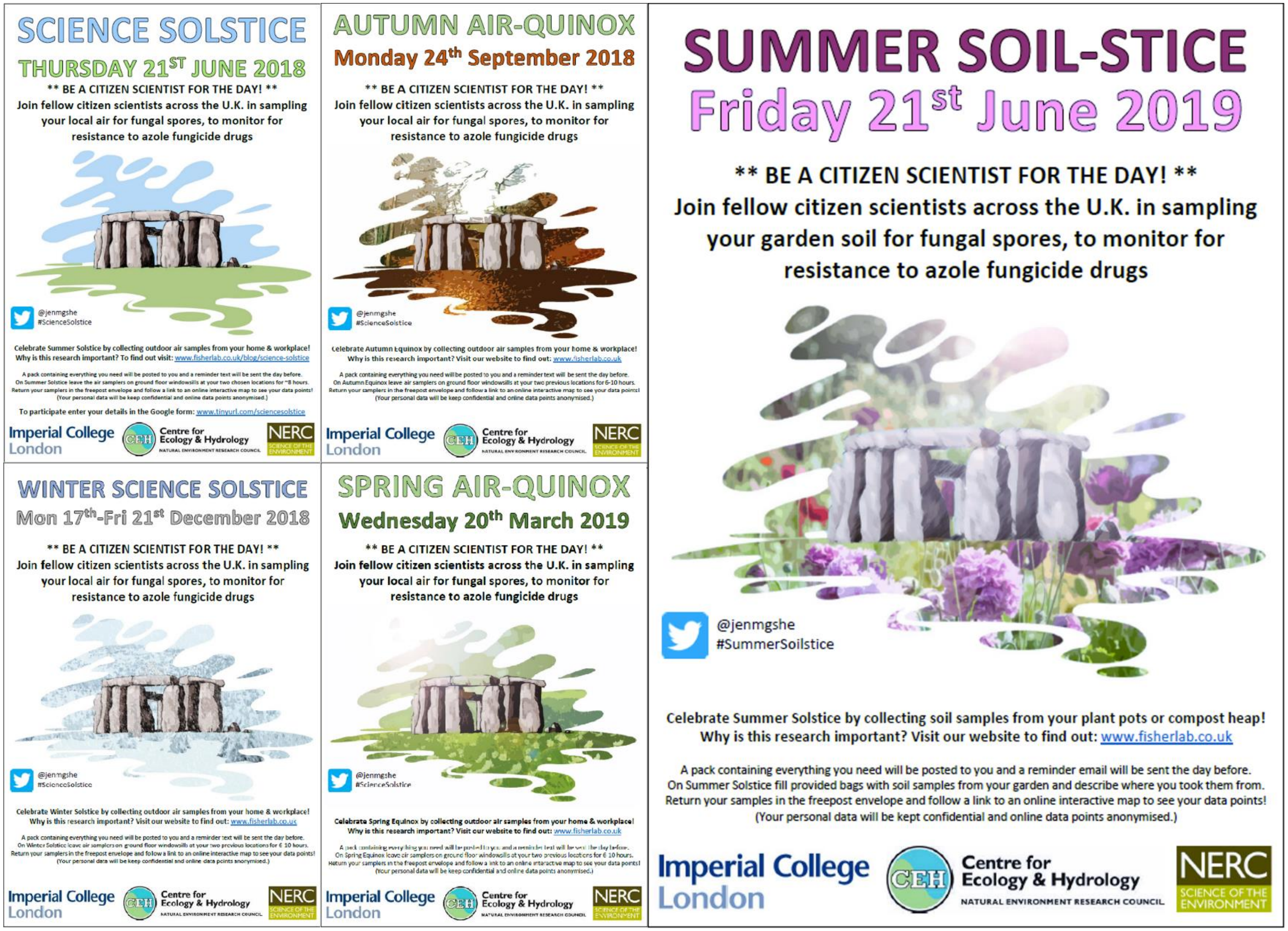

Fig. S1.

Posters displayed on social media to advertise four air sampling rounds that took place on solstice and equinox dates between June 2018 and June 2019 (left) and the subsequent soil sampling round that took place in March 2019 (right).

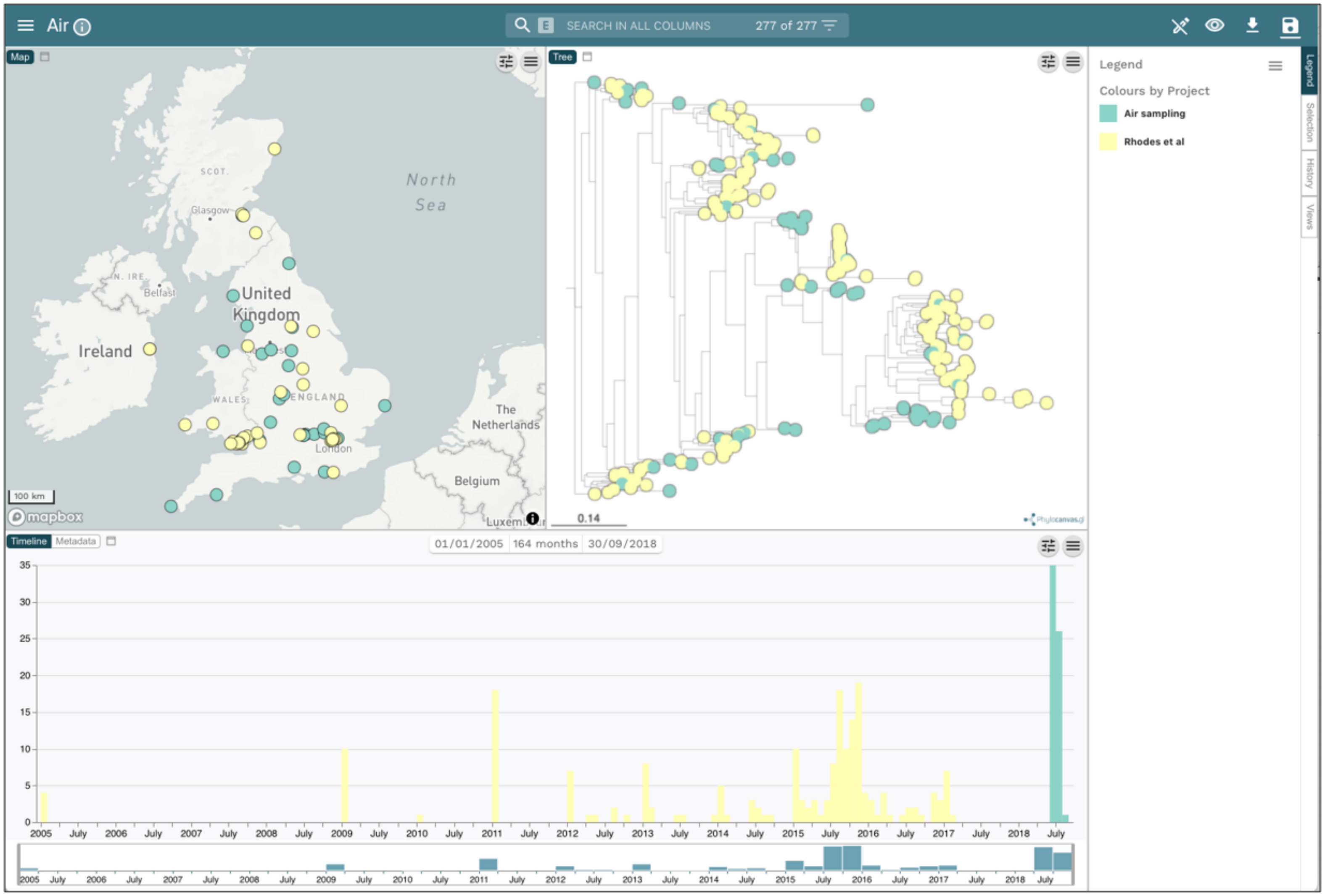

Fig. S2.

Sampling locations and maximum-likelihood phylogenies constructed from whole-genome data of 62 air-sampled *Aspergillus fumigatus* isolates and 215 environmental and clinical *A. fumigatus* isolates collected in the UK between 2005 and 2017 visualized using Microreact (https://microreact.org/project/6NMrDobYGZnhmYnMSsHhC5-air).

Sample (prefix for sampling round)	County, north to south	ITZ	VCZ	PCZ	ISZ	TebuCheck result (mg/L)	*cyp51A* polymorphisms
SS18-084-02	Edinburgh	**16**	**2**	0.25	**4**	16	TR$_{34}$/L98H
SS18-251-01	Northumberland	**16**	**2**	0.25	**2**	-	TR$_{34}$/L98H
SS18-073-01	Dumfries & Galloway	0.25	**2**	0.12	0.5	16	*A. lentulus*
SS18-074-02	Cumbria	**16**	**2**	**0.5**	**2**	16	TR$_{34}$/L98H
SS18-068-01	Lancashire	**16**<br>**16**	**2**<br>**2**	0.25<br>0.25	**2**<br>**2**	8<br>6	TR$_{34}$/L98H<br>TR$_{34}$/L98H
SS18-007-02	West Yorkshire	**16**	**2**	0.25	**2**	16	TR$_{34}$/L98H
SS18-381-02	Cheshire	**16**	**2**	0.25	**2**	-	TR$_{34}$/L98H/N479S
SS18-202-02	South Yorkshire	**16**	**4**	0.25	**2**	-	TR$_{34}$/L98H
SS18-403-01	Conway	**16**	**2**	0.25	**2**	-	WT
SS18-075-01	Cheshire	**16**	**4**	**0.5**	**4**	8	TR$_{34}$/L98H
SS18-257-01	Derbyshire	**16**	**2**	**0.5**	**2**	-	TR$_{34}$/L98H
SS18-261-02	West Midlands	**16**<br>0.12	**2**<br>0.5	**0.5**<br>0.06	**2**<br>0.5	-<br>-	TR$_{34}$/L98H<br>WT
SS18-278-02	West Midlands	**16**	1	**0.5**	**2**	-	TR$_{34}$/L98H
SS18-085-01	Worcestershire	**16**	**2**	0.25	**4**	16	TR$_{34}$/L98H
SS18-139-01	Suffolk	**16**	**2**	0.25	**4**	-	TR$_{34}$/L98H
SS18-177-02	Gloucestershire	**16**	**2**	0.25	**2**	-	WT
SS18-143-01	Hertfordshire	**16**	**2**	0.25	**4**	-	TR$_{34}$/L98H
SS18-082-01	Hertfordshire	**16**<br>**16**<br>**16**<br>**16**	**4**<br>**4**<br>**2**<br>**2**	**0.5**<br>**0.5**<br>0.25<br>0.25	**4**<br>**4**<br>**4**<br>**2**	16<br>8<br>6<br>8	TR$_{34}$/L98H<br>TR$_{34}$/L98H<br>TR$_{34}$/L98H<br>TR$_{34}$/L98H
SS18-056-02	Buckinghamshire	**16**<br>**16**<br>**16**<br>**16**	1<br>**2**<br>**2**<br>**2**	**0.5**<br>0.25<br>0.25<br>0.25	**2**<br>**2**<br>**2**<br>**2**	6<br>6<br>8<br>8	TR$_{34}$/L98H<br>TR$_{34}$/L98H<br>TR$_{34}$/L98H<br>TR$_{34}$/L98H
SS18-005-02	Oxfordshire	**16**	**16**	**0.5**	**16**	16	TR$_{46}$/Y121F/T289A
SS18-013-01	Oxfordshire	**16**	**2**	0.25	**2**	8	TR$_{34}$/L98H
SS18-040-01	Oxfordshire	**16**	**2**	**0.5**	**2**	8	TR$_{34}$/L98H
SS18-375-02	Oxfordshire	0.25	1	0.06	1	-	*A. lentulus*
SS18-340-01	London	**16**<br>**16**	**2**<br>**2**	0.25<br>0.25	**2**<br>**2**	-<br>-	TR$_{34}$<br>TR$_{34}$/L98H
SS18-423-01	Bristol	0.12	0.12	0.03	0.25	-	WT
SS18-304-01	Hampshire	**16**	**2**	0.25	**2**	-	TR$_{34}$/L98H
SS18-193-02	West Sussex	**16**<br>0.12	1<br>0.25	**0.5**<br>0.03	**2**<br>0.5	-<br>-	TR$_{34}$/L98H<br>WT
SS18-248-01	Hampshire	**16**	**16**	**0.5**	**16**	-	*A. lentulus*
SS18-204-01	Devon	**16**<br>**16**<br>**16**	**2**<br>**2**<br>**2**	**0.5**<br>0.25<br>**0.5**	**2**<br>**2**<br>**2**	-<br>-<br>-	TR$_{34}$/L98H<br>TR$_{34}$/L98H<br>TR$_{34}$/L98H

SS18-285-01	Cornwall	**16**	**2**	0.25	**4**	-	WT
AE18-081-01	Fife	**16**	**4**	0.25	**2**	-	TR$_{34}$/L98H
AE18-026-01	Cumbria	**16**	**4**	**0.5**	**4**	-	TR$_{34}$/L98H/D481N
AE18-098-02	Lancashire	**16**	0.5	**0.5**	**4**	-	WT
		**16**	**2**	0.25	**2**		WT
AE18-098-01	Lancashire	**16**	**2**	0.25	**4**	-	D262Y
		**16**	**2**	0.25	**2**	-	WT
		**16**	**2**	0.25	**2**	-	WT
		**16**	**2**	0.25	**2**	-	WT
		**16**	**2**	0.25	**2**	-	WT
		**16**	1	0.25	**2**	-	WT
		0.25	0.25	0.06	0.25	-	WT
		**16**	**2**	0.25	**2**	-	TR$_{34}$/L98H
		**16**	**2**	0.25	**2**	-	WT
		**16**	**2**	0.25	**2**	-	WT
AE18-016-02	Lancashire	**16**	**2**	0.25	**2**	-	TR$_{34}$/L98H
AE18-060-01	West Midlands	**16**	**2**	0.25	**2**	-	TR$_{34}$/L98H
AE18-125-01	Northamptonshire	**16**	1	0.25	**2**	-	TR$_{34}$/L98H
AE18-124-01	Essex	**16**	**2**	**0.5**	**2**	-	TR$_{34}$/L98H
AE18-037-01	Hertfordshire	**16**	**2**	0.25	1	-	*A. lentulus*
AE18-082-02	Hertfordshire	0.25	0.5	0.06	0.5	-	WT
AE18-211-01	Oxfordshire	**16**	1	0.25	**2**	-	TR$_{34}$/L98H
AE18-225-02	Cardiff	0.06	0.13	0.03	0.12	-	*A. nidulans*
AE18-064-01	London	**16**	**2**	0.25	**2**	-	TR$_{34}$/L98H
AE18-187-01	Wiltshire	**16**	1	0.25	**2**	-	TR$_{34}$/L98H
		**16**	**2**	**0.5**	**4**	-	TR$_{34}$/L98H
		0.12	0.25	0.03	0.25	-	WT
WS18-061-01	Dumfries & Galloway	0.12	0.12	0.03	0.25	-	*A. lentulus*
WS18-061-02	Dumfries & Galloway	0.25	1	0.12	1	-	*A. lentulus*
WS18-134-02	Cumbria	**16**	1	0.25	**2**	-	TR$_{34}$/L98H
		**16**	**4**	**0.5**	**8**	-	TR$_{34}$/L98H
WS18-060-01	West Yorkshire	ND	ND	ND	ND	-	TR$_{34}$/L98H
WS18-028-02	Lancashire	**16**	1	0.25	**2**	-	TR$_{34}$/L98H
WS18-153-01	Merseyside	**16**	1	0.25	**2**	-	TR$_{34}$/L98H
		**16**	1	0.25	**2**	-	TR$_{34}$/L98H
WS18-119-02	Nottinghamshire	**16**	**2**	0.25	**4**	-	TR$_{34}$/L98H
WS18-045-01	Lincolnshire	**16**	1	0.25	**2**	-	TR$_{34}$/L98H
WS18-045-02	Lincolnshire	**16**	1	0.25	**2**	-	TR$_{34}$/L98H
WS18-040-01	Oxfordshire	**16**	**2**	0.25	**2**	-	TR$_{34}$/L98H
		0.06	0.25	0.03	0.5	-	D262Y
WS18-161-01	Mid Glamorgan	0.5	1	0.12	1	-	*A. lentulus*
WS18-125-01	Surrey	**16**	1	0.25	**2**	-	TR$_{34}$/L98H
SA19-182-02	Dundee	0.12	0.12	0.03	0.25	-	WT

SA19-178-01	Londonderry	0.06	0.12	0.03	0.25	-	*A. nidulans*
SA19-122-01	West Yorkshire	**16**<br>**16**	1<br>1	0.25<br>0.25	1<br>1	-<br>-	*A. lentulus*<br>*A. lentulus*
SA19-184-01	Cheshire	0.06	0.25	0.03	0.25	-<br>-	S46F/R66W/<br>M172V/E427K
SA19-050-01	Staffordshire	**16**	1	0.25	**2**	-	D262Y
SA19-051-01	Cambridgeshire	**16**	1	0.25	**2**	-	WT
SA19-170-01	Powys	**16**	**16**	0.25	**16**	-	TR_{46}/Y121F/T289A
SA19-207-02	Northamptonshire	0.25	**16**	0.12	**16**	-	TR_{46}/Y121F/T289A
SA19-176-01	Suffolk	**16**<br>**16**<br>**16**<br>0.06<br>0.12<br>0.06<br>0.06<br>0.06	1<br>1<br>**2**<br>0.25<br>0.25<br>0.25<br>0.25<br>0.25	0.25<br>0.25<br>0.25<br>0.03<br>0.03<br>0.03<br>0.03<br>0.03	**2**<br>**2**<br>**2**<br>0.25<br>0.5<br>0.25<br>0.25<br>0.25	-<br>-<br>-<br>-<br>-<br>-<br>-<br>-	WT<br>TR_{34}/L98H<br>WT<br>WT<br>WT<br>WT<br>WT<br>WT
SA19-154-02	Carmarthenshire	**16**<br>**16**	**16**<br>1	0.25<br>0.25	**8**<br>**2**	-<br>-	TR_{46}/Y121F/T289A<br>TR_{34}/L98H
SA19-001-03	Oxfordshire	**16**	1	0.25	**2**	-	TR_{34}/L98H
SA19-208-01	Oxfordshire	**16**	**2**	0.25	**4**	-	TR_{34}/L98H/V486I
SA19-063-01	Essex	0.12<br>0.5	0.25<br>0.5	0.03<br>0.03	0.25<br>0.5	-<br>-	*A. lentulus*<br>WT
SA19-004-01	Oxfordshire	0.25	0.25	0.03	0.5	-	WT
SA19-120-01	Oxfordshire	**16**	**2**	0.25	**2**	-	TR_{34}/L98H
SA19-078-02	Mid Glamorgan	**16**	**2**	0.25	**2**	-	WT
SA19-162-01	Wiltshire	**16**<br>**16**	**16**<br>**16**	0.25<br>0.25	**16**<br>**16**	-<br>-	TR_{46}/Y121F/T289A<br>TR_{46}/Y121F/T289A

Table S1.

Details of the 111 UK tebuconazole-resistant isolates including the unique identifier for the air sample they were grown from, the county from which the sample was collected, their minimum inhibitory concentrations (MICs) for itraconazole (ITZ), voriconazole (VCZ), posaconazole (PCZ) and isavuconazole (ISZ), Tebucheck result and *cyp51A* polymorphisms. Sample prefix indicates air sampling round that sample was collected in: SS18=21st June 2018, AE18=24th September 2018, WS18=21st December 2018, SA19=20th March 2019. Resistant MICs are highlighted in bold and isolates subsequently found not to be *Aspergillus fumigatus* are highlighted in red. WT=wild-type. ND=not done. Note that all isolates were originally screened for resistance to tebuconazole on SDA plates containing 6 mg/L tebuconazole, and only a subset of isolates were further tested using the Tebucheck protocol.

County, north to south	Air sampling																Soil sampling			
	Summer 2018				Autumn 2018				Winter 2018				Spring 2019				Summer 2019			
	1		2		1		2		1		2		1		2		1		2	
	A	R	A	R	A	R	A	R	A	R	A	R	A	R	A	R	A	R	A	R
Inverness-shire					0	0			2	0			1	0			30	0	5	0
Fife	1	0			3	0			0	0			1	0			0	0	0	0
Midlothian	2	0	0	0													0	0	30	3
**Dumfriesshire**	**0**	**0**			**0**	**0**			**7**	**1**			**0**	**0**			**1**	**1**		
Lancashire	3	0	5	0									0	0			5	0	3	3
Lancashire													0	0			50	0	0	0
West Yorkshire					1	0	0	0	34	1	35	0	0	0	0	0	2	0	0	0
Lancashire									8	0	7	0	0	0	0	0	9	0	12	0
Greater Manchester	1	0			0	0			0	0			0	0			4	0	2	0
Greater Manchester													0	0	0	0	11	6		
Cheshire													0	0			0	0	0	0
Cheshire													1	0			0	0	0	0
Cheshire	2	0	0	0	2	0	0	0	2	0	1	0	0	0	0	0	22	0	50	17
Staffordshire					0	0			1	0			1	1			0	0	0	0
Norfolk													1	0			3	0	0	0
West Midlands					2	0			0	0			0	0	0	0	0	0	0	0
West Midlands	7	0	8	2	3	0	2	0	0	0	0	0	0	0	0	0	30	0	0	0
West Midlands	0	0	1	0													3	0	22	0
Northamptonshire					1	0	1	0	1	0	0	0	1	0	0	0	0	0	2	0
Gloucestershire													0	0	0	0	30	8	30	3
Hertfordshire					3	0			11	0			6	0	1	0	50	1	0	0
Hertfordshire													1	0			0	0	6	0
Breconshire	0	0			0	0											0	0	9	0
Oxfordshire	0	0			0	0							0	0			30	30	6	0
Buckinghamshire	0	0	1	0	0	0	0	0	0	0	0	0	0	0	0	0	0	0	50	19
Essex									0	0			0	0			0	0	0	0
Buckinghamshire	2	0			1	0			0	0			0	0			3	0	30	3
Oxfordshire	1	0															30	1	30	0
Oxfordshire	4	0			1	1			1	0			0	0			5	0	0	0
**Oxfordshire**	**0**	**0**	**1**	**0**	**3**	**0**	**1**	**0**	**2**	**0**	**1**	**0**	**1**	**1**	**1**	**0**	**25**	**8**	**25**	**2**
Oxfordshire	0	0			0	0			0	0			0	0			30	2	30	6
Greater London	7	0	5	0	2	0	5	0	3	0	0	0					2	0	17	0
Greater London	1	0	0	0	1	0							0	0			19	0	2	0
Buckinghamshire									0	0	3	0	1	0	1	0	4	0	12	0
Greater London	1	0			0	0							4	0			16	1	8	0
Greater London	0	0															25	9	30	5
**Greater London**	**2**	**0**	**1**	**0**	**1**	**1**	**0**	**0**	**0**	**0**	**0**	**0**	**0**	**0**	**0**	**0**	**30**	**1**	**30**	**2**
Hampshire	2	0			1	0			0	0			0	0			30	0	4	0
Wiltshire	0	0	0	0	0	0	1	0	0	0	1	0	0	0	1	0	30	0	0	0
Somerset					1	0	1	0					0	0	1	0	0	0	30	3
Dorset					0	0			0	0			0	0			0	0	0	0
Hampshire	5	1			1	0			1	0			3	0			0	0	0	0
Dorset	5	0	0	0	0	0	0	0	0	0	0	0	1	0	1	0	12	0	30	1
Cornwall	0	0			2	0			2	0			0	0			3	0	3	0

Table S2.

Details of 46 UK locations from which both an air and soil sample were collected, including the county and the number of *A. fumigatus* (A) and tebuconazole-resistant *A. fumigatus* (R) colonies grown from each sample. Samples that grew tebuconazole-resistant *A. fumigatus* are highlighted in red and locations that grew tebuconazole-resistant *A. fumigatus* from both air and soil samples are highlighted in bold.
